# Bile Acid Metabolic Profiles and Their Correlation with Intestinal Epithelial Cell Proliferation and Barrier Integrity in Suckling Piglets

**DOI:** 10.3390/ani14020287

**Published:** 2024-01-17

**Authors:** Min Zhu, Chong Lin, Kaimin Niu, Yichun Liu, Weirong Zeng, Ruxia Wang, Xiongchang Guo, Zhenya Zhai

**Affiliations:** 1 Laboratory of Animal Genetics, Breeding and Reproduction in the Plateau Mountainous Region, Ministry of Education, College of Animal Science, Guizhou University, Guiyang 550025, China; mzhudky@gzu.edu.cn; 2 Jiangxi Functional Feed Additive Engineering Laboratory, Institute of Biological Resource, Jiangxi Academy of Sciences, Nanchang 330096, China; lc344635040@foxmail.com (C.L.); niulele88@126.com (K.N.); bngleliu@foxmail.com (Y.L.); zwr2282749541@foxmail.com (W.Z.); wangruxia317@126.com (R.W.); gxc411930833@foxmail.com (X.G.)

**Keywords:** bile acid metabolism, suckling piglets, FXR, TGR5, cell proliferation, intestinal barrier function

## Abstract

**Simple Summary:**

Bile acids (BAs) play an essential role in nutrient absorption and intestinal development for piglets. However, there remains a lack of clarity on the metabolic changes in BAs in piglets after birth and their relationship with intestinal development. This study found significant changes in BA metabolism in piglets after birth. In comparison with newborn piglets (NPs), the total serum BA content was increased for suckling piglets (SPs). Furthermore, the metabolic changes in BAs were highly correlated with the expression of genes related to BA receptors and the regulation of intestinal proliferation and barrier integrity, thereby laying the groundwork for the nutritional regulation of suckling piglets. This can also provide more insights into how the economic benefits of pig farming could be increased.

**Abstract:**

Bile acids (BAs) are crucial for maintaining intestinal epithelial homeostasis. However, the metabolic changes in BAs and the communication between intestinal epithelial cells (IECs) in infants after birth remain unclear. This study aims to elucidate the BA profiles of newborn piglets (NPs) and suckling piglets (SPs), and to investigate their regulatory effects on IEC proliferation and barrier integrity, as well as the potential underlying mechanisms. In this study, compared with NPs, there were significant increases in serum triglycerides, total cholesterol, glucose, and albumin levels for SPs. The total serum BA content in SPs exhibited an obvious increase. Moreover, the expression of BA synthase cytochrome P450 27A1 (CYP27A1) was increased, and the ileal BA receptor Takeda G-coupled protein receptor 5 (TGR5) and proliferation marker Ki-67 were upregulated and showed a strong positive correlation through a Spearman correlation analysis, whereas the expression of farnesoid X receptor (FXR) and occludin was markedly downregulated in SPs and also revealed a strong positive correlation. These findings indicate that the increased synthesis and metabolism of BAs may upregulate TGR5 and downregulate FXR to promote IEC proliferation and influence barrier function; this offers a fresh perspective and evidence for the role of BAs and BA receptors in regulating intestinal development in neonatal pigs.

## 1. Introduction

The small intestine is the most critical organ for the digestion and absorption of nutrients in animals. At the same time, small-intestinal epithelial cells act as a physical barrier, serving as a site for microbial colonization and providing resistance against external pathogen invasion. The development and maturation of the intestinal tract in suckling piglets are of vital importance for enhancing their survival rate and subsequent production performance, profoundly impacting the production efficiency of the pig farming industry [1]. The intestinal development of newborn piglets commences at the embryonic stage; however, the intestinal structure and function of newborn piglets remain imperfect even after birth [2]. Research has shown that piglets exhibit a reduced villus height and goblet cell numbers and downregulated protein expression of tight junction proteins in the ileum for 14 days after birth [3,4]. This suggests that the first 14 days after birth may be a critical period for intestinal development, raising the question of which factors and regulatory mechanisms govern intestinal development during this time.

Bile acids (BAs) are abundant small-molecule metabolites in the intestine that not only aid in the absorption of nutrients (such as fats) but also regulate metabolism (including maintaining the homeostasis of triglycerides, cholesterol, and glucose) and body development as essential signaling molecules [5,6]. Recent studies have demonstrated that BAs play critical roles in shaping intestinal development, intestinal barrier function, and immunological modulation [6,7,8]. Supplementing the diet with a mixed bile acid preparation can significantly improve the structure of the colonic intestinal barrier and gut microbiota in piglets with intrauterine growth restriction (IUGR), thereby maintaining their intestinal health [9]. Additionally, research has shown that ursodeoxycholic acid can inhibit inflammation by regulating the polarization of M2 macrophages, thereby potentially improving intestinal health in IUGR piglets [10]. Chenodeoxycholic acid (CDCA) can alleviate lipopolysaccharide (LPS)-induced IPEC-J2 cell barrier disruption [11]. Increases in the levels of BAs such as lithocholic acid (LCA) in the intestine contribute to the remission and recovery of inflammatory bowel disease (IBD) in both mice and humans [8]. However, LCA and deoxycholic acid (DCA) have been found to inhibit proliferation and induce intestinal epithelial injury in piglets [12]. Similarly, studies have reported that hyodeoxycholic acid (HDCA) inhibits the proliferation and differentiation of IPEC-J2 cells and intestinal organoids [13,14]. Similarly, compared to normal piglets, the levels of LCA and dehydrocholic acid (DHCA) in the intestines of IUGR piglets were significantly increased, which may be the reason for the growth inhibition of IUGR piglets [15]. Due to the diverse range of BA types, the regulatory effects of different BAs on the intestine may be species- or tissue-specific or related to the physiological or pathological state of the body. Nevertheless, what changes occur in BA metabolism in piglets after birth and whether BAs can regulate intestinal development in young animals remain to be explored.

Farnesoid X receptor (FXR) and Takeda G-coupled protein receptor 5 (TGR5) are the two most critical BA receptors in the gut; however, they exhibit distinct regulatory effects on intestinal cell bioactivities. Initial studies on FXR primarily focused on regulating the “liver−gut circulation” of BAs. When BAs activate FXR in the intestinal lumen, it promotes the secretion of fibroblast growth factor 19 (FGF-19) into the bloodstream, consequently inhibiting BA synthesis [6]. Recent research has revealed that FXR activation can suppress cell proliferation in weaned piglets while mitigating LPS-induced intestinal barrier damage [11,13,16]. In contrast to FXR, TGR5 serves as a membrane receptor for BAs, and its functions in piglet models have not been reported. Nevertheless, in humans and mice, the absence of TGR5 expression can result in intestinal inflammation, inhibit intestinal stem cell proliferation and differentiation, and disrupt intestinal epithelial homeostasis [17,18]. As such, FXR and TGR5 play crucial roles in maintaining intestinal health. However, the manner in which their expression levels change after piglets’ birth and their potential relationship with intestinal development remain uncertain.

This study aimed to investigate the developmental changes in BA profiles in piglets throughout intestinal development during early lactation. Moreover, the connections between critical BAs and intestinal cells’ proliferation and barrier expression were analyzed. The findings of this study are anticipated to provide valuable insights into the growth and intestinal health of young piglets and offer guidance for the application of BAs in the pig farming industry.

## 2. Materials and Methods

### 2.1. Ethics Approval

The animal experiment was approved by the Animal Care Committee of the Institute of Biological Resource, Jiangxi Academy of Sciences. All protocols were carried out under the guidelines of the Institute of Biological Resource on Animal Care, Jiangxi Academy of Sciences (No. IBR-2021-15).

### 2.2. Animals and Study Design

On day 95 of gestation, 12 sows (parity, similar body condition, Landrace × Large White) were selected and fed until the birth of their piglets. The sows’ parity was determined to be 7.56 ± 0.4. The basal diets provided for the sows during gestation or lactation were formulated to meet their nutritional requirements (NRC 2012), with the ingredients detailed in Table 1. Their body condition was assessed based on backfat thickness, as measured using an ultrasonic device (PIGLOG105, SFAK Technology, Helver, Denmark) at 65 mm on both sides of the dorsal midline of the last rib (P2). The backfat thickness data are presented in Table 2.

Following the birth of their piglets, the sows were fed until day 14 of lactation. During gestation, the sows were fed twice daily (6:30 and 14:30), receiving 1.5 kg of feed each time (3 kg in total). The average total feed intake per sow during gestation was 60.30 ± 1.48 kg. During the lactation period, the sows were fed 1.5 kg of diet on day 1 and 2.5 kg on day 2. The feed amounts were increased by 0.5 kg each day from day 3 to day 6, with the average total feed intake per sow being 19.03 ± 0.02 kg. From day 7 to day 14, the sows were fed ad libitum, resulting in an average total feed intake per sow of 59.02 ± 0.23 kg. Table 2 displays the average body weight (ABW), litter weight, and average daily gain (ADG).

On the day of birth, 12 male piglets with body weights close to the average were selected from 12 sows (1 piglet per sow) and slaughtered for sampling (newborn piglets). In contrast, another 12 piglets were nursed by the sows until 14 days of age (suckling piglets) and then slaughtered. Sow milk served as the sole nutritional source for the suckling piglets. During the 7–14 days of lactation, the equivalent feed intake of sows for suckling piglets was weighed, and the average daily feed intake was calculated (5.15 ± 0.15 kg/d). All sows and piglets had free access to water.

### 2.3. Sample Preparation

Serum, liver, and intestinal samples were collected from piglets on the day of birth and 14 days after birth. Newborn piglet samples were collected within 1 h of birth, while samples from 14-day-old piglets were collected after fasting for 8 h. Following an intraperitoneal injection of anesthesia with 2% pentobarbital sodium (45 mg/kg body weight), blood samples were drawn into 1.5 mL centrifuge tubes (germ-RNase- and DNase-free) from the anterior vena cava. Serum samples were then obtained via centrifugation at 900 g for 10 min at 4 °C and stored at −80 °C. Liver samples (1 g each) were collected near the gallbladder from each piglet. Intestinal mucosal samples were obtained from a location approximately 10 cm from the end of the ileum. Both the liver and intestinal samples were flash-frozen in liquid nitrogen and stored at −80 °C until PCR or other tests were performed.

### 2.4. Detection of Serum Biochemical Parameters and Hepatic Lipids

As for the concentrations of serum total protein (TP), albumin (ALB), alanine aminotransferase (ALT), aspartate transaminase (AST), alkaline phosphatase (ALP), blood urea nitrogen (BUN), glucose (GLU), total cholesterol (TC), triglyceride (TG), low-density lipoprotein cholesterol (LDL-C), high-density lipoprotein cholesterol (HDL-C), and total bile acids (TBAs), they were measured with the assistance of an automated biochemistry analyzer (Synchron CX Pro, Beckman Coulter, Fullerton, CA, USA) and commercial kits (Chemlin, Beijing, China). The hepatic total cholesterol (TC), triglyceride (TG), and TBA concentrations were analyzed using the commercial assay kits supplied by Nanjing Jiancheng Bioengineering Institute (Nanjing, Jiangsu, China). The contents of the corresponding indicators were corrected using total protein.

### 2.5. Real-Time Quantitative PCR (RT-qPCR)

The critical genes were assessed against BA receptors, BA synthesis, BA transport, and intestinal development. Total RNA was extracted from the frozen ileum and liver tissues using a MagZol Reagent (Magen, Guangzhou, China). The concentration of RNA was estimated using a NanoDrop 2000C spectrophotometer (Thermo Fisher Scientific, Waltham, MA, USA). The cDNA was reverse-transcribed from 1000 ng of RNA using a reverse-transcription kit (CWBIO, Jiangsu, China) according to the manufacturer’s instructions. The primer sequences (Table S1) were designed and synthesized by a commercial company (Sangon Biotech, Shanghai, China). The quantitative real-time PCR was performed on a CFX96 Real-Time PCR Detection System (Bio-Rad, Hercules, CA, USA) with the assistance of the qPCR SYBR Green Master Mix kits (Yeassen, Shanghai, China). The relative levels of gene expression were normalized to the housekeeping gene β-actin and then calculated using the comparative CT method (2^−ΔΔCt^) [19].

### 2.6. Determination of BA Composition in Serum

The BA concentrations in serum were measured utilizing ultrahigh-performance liquid chromatography–tandem secondary mass spectrometry (UHPLC-MS/MS) and according to the protocol described in our previous study [18]. In brief, the serum was mixed with an extract solvent (acetonitrile/methanol, 1:1, containing 0.1% formic acid and an isotopically labeled internal standard mixture), vortexed for 30 s, sonicated for 10 min in an ice-water bath, incubated at −40 °C for 1 h, and centrifuged at 12,000 g and 4 °C for 15 min. Then, the prepared samples were detected using a Q-Exactive Focus mass spectrometer (Thermo Fisher Scientific), in combination with an Agilent 1290 Infinity series UHPLC System (Agilent Technologies, Santa Clara, CA, USA) equipped with a Waters ACQUITY UPLC BEH C18 column (150 × 2.1 mm, 1.7 μm, Waters). The typical ion source parameters were as follows: spray voltage = +3500/−3100 V; sheath gas (N2) flow rate = 40; aux gas (N2) flow rate = 15; sweep gas (N2) flow rate = 0; aux gas (N2) temperature = 350 °C; capillary temperature = 320 °C. All detected BAs were classified into primary BAs (PBAs), secondary BAs (SBAs), free BAs (FBAs), and conjugated BAs (CBAs) according to their synthetic sources and properties.

### 2.7. Statistical Analysis

All data were analyzed using SPSS 25.0 (IBM, SPSS, Chicago, IL, USA) and expressed as the mean ± SEM. The Mann–Whitney U test was conducted to determine the statistical differences. A correlation analysis was carried out using Spearman’s test. The differences were treated as statistically significant when *p* < 0.05. GraphPad Prism 9 (GraphPad Software Inc., San Diego, CA, USA) was used to generate the charts.

## 3. Results

### 3.1. Serum Biochemical Parameters of Newborn and Suckling Piglets

The serum levels of TP, ALB, ALT, AST, and GLU were analyzed and are presented in Table 3. Compared with those in newborn piglets, the serum levels of TP, ALB, ALT, AST, and GLU in suckling piglets significantly increased (*p* < 0.05), while the ALP levels decreased (*p* < 0.01). No significant difference was observed in BUN content (*p* > 0.05).

### 3.2. Lipid and Total BA Contents in Sera and Livers of Newborn and Suckling Piglets

As anticipated, the lipid contents (TG, TC, LDL-C, HDL-C, and TBA) in the sera of suckling piglets (SPs) were significantly increased compared to those in newborn piglets (NPs) (Table 4; *p* < 0.05). In the liver, the TG content of the SPs was significantly lower than that of the NPs (*p* = 0.044, Table 4). However, no significant differences were found in the TC and TBA contents (*p* > 0.05, Table 4).

### 3.3. Contents and Composition of BAs in the Sera of Newborn and Suckling Piglets

The aforementioned results indicate that the serum TBAs were significantly upregulated. Furthermore, the contents of BAs in the piglets’ sera were determined using UHPLC-MS/MS. As shown in Table 5, the contents of most BAs in the SP sera increased compared to the NP sera. The contents of hyocholic acid (HCA), glycine-conjugated HCA (GHCA), HDCA, chenodeoxycholic acid (CDCA), taurine-conjugated HDCA (THDCA), cholic acid (CA), deoxycholic acid (DCA), and lithocholic acid (LCA) in the SP sera were significantly higher than those in the NP sera (*p* < 0.05). Notably, 12-DHCA, 3-DHCA, α-MCA, UDCA, 7-KHCA, and isoLCA were not detected in the NP sera; however, trace amounts were observed in the SP sera. In contrast, the contents of TCDCA, GCDCA, GCA, and TCA in the SP sera decreased compared to the NP sera, with no statistical significance between the groups (*p* > 0.05).

The composition of serum BAs in the piglets was further analyzed based on BA classification and the proportion of each BA in piglet sera to demonstrate the differences in serum composition between NPs and SPs. As shown in Table 5, the serum primary BA (PBA) and secondary BA (SBA) contents in the SPs were 6.7 and 6.9 times higher than those in the NPs, respectively, indicating a significant increase compared to the NPs (*p* < 0.05). Similarly, the serum contents of FBAs in the SPs were significantly increased compared to the NPs (*p* < 0.05).

**Figure 1:**
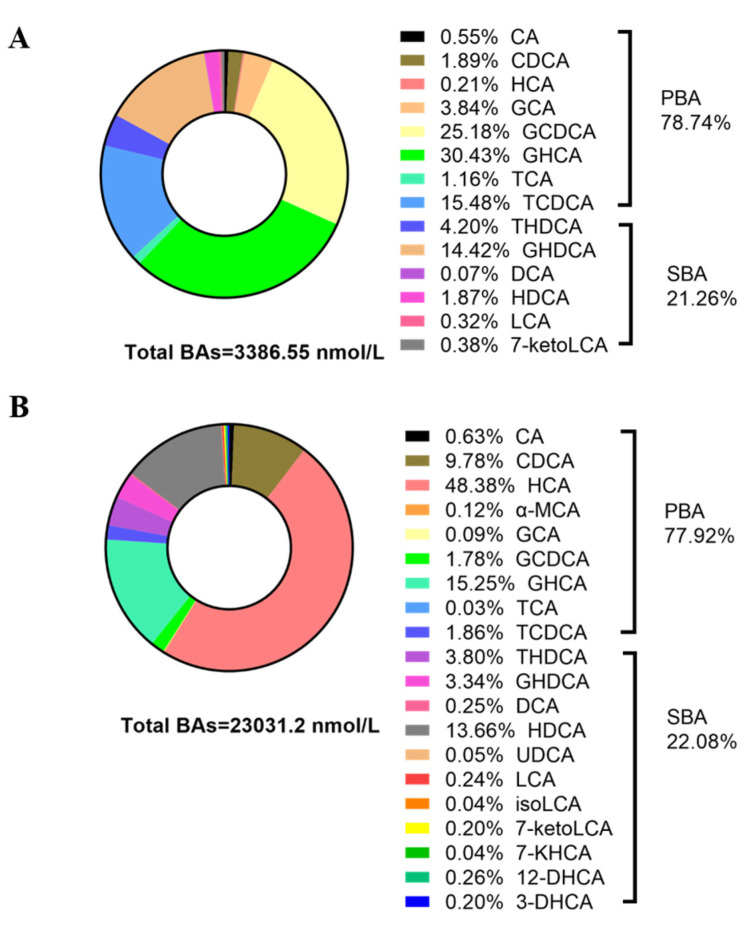
Bile acid composition in newborn piglets (NPs) and suckling piglets (SPs): (**A**) the serum BA content profile in NPs (*n* = 6/group). (**B**) The serum BA content profile in SPs (*n* = 6/group). NPs: newborn piglets, SPs: 14-day-old suckling piglets; *n* = 6 replicates per group.

To better illustrate the metabolic changes in BAs in piglets after birth, the proportions of different BAs were also calculated (Figure 1A,B). Compared to newborn piglets, the types of BAs in SP serum were more abundant, with 20 types of BAs in SP serum and only 14 types in NP serum. The proportions of primary BAs (PBAs, 77.92–78.74%) and secondary BAs (SBAs, 21.26–22.08%) remained relatively consistent. Furthermore, the serum of the NPs primarily consisted of GHCA (30.43%), GCDCA (25.18%), TCDCA (15.48%), and GHDCA (14.42%), whereas the serum of the SPs predominantly contained HCA (48.38%), GHCA (15.25%), HDCA (13.66%), and CDCA (9.78%).

### 3.4. Differences in BA Metabolism between Newborn Piglets and Suckling Piglets

**Figure 2:**
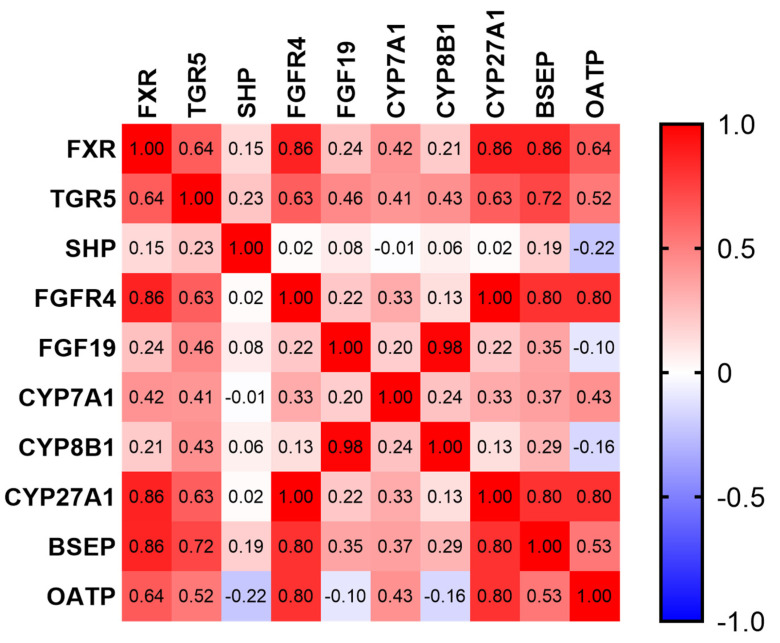
The correlation analysis between liver mRNA expression and bile acids in newborn piglets (NPs) and suckling piglets (SPs). Correlation analysis between bile acid metabolism and receptor and transport gene expression. Spearman’s method was used to analyze the correlation between variables (*n* = 12). FXR: farnesoid X receptor, TGR5: Takeda G-coupled protein receptor 5, SHP: small heterodimer partner, FGFR4: fibroblast growth factor receptor 4, FGF19: fibroblast growth factor 19, CYP7A1: cytochrome P450 family 7 subfamily A member 1, CYP8B1: cytochrome P450 family 8 subfamily B member 1, CYP27A1: cytochrome P450 family 27 subfamily A member 1, BSEP: bile salt output pump, OATP: organic anion transport peptide.

To better analyze the metabolic processes of BAs in the livers and intestines of newborn and suckling piglets, the mRNA expression levels of BA synthase, hepatic–intestinal circulation, and transport-related genes in the liver and ileum were detected using RT-qPCR. From the perspective of BA synthesis, the mRNA level of cytochrome P450 family 27 subfamily A member 1 (CYP27A1) in the livers of suckling piglets (SPs) increased significantly (Table 6; *p* < 0.05), while no significant difference in the expression of cytochrome P450 family 7 subfamily A member 1 (CYP7A1) and cytochrome P450 family 8 subfamily B member 1 (CYP8B1) (*p* > 0.05) were observed. The BA hepatic–intestinal circulation-related gene expression is also presented in Table 6. Compared to newborn piglets (NPs), the mRNA levels of TGR5 and fibroblast growth factor receptor (FGFR) 4 in the liver significantly increased, while farnesoid X receptor (FXR), small heterodimer partner (SHP), and fibroblast growth factor 19 (FGF19) showed no significant differences in the liver (*p* > 0.05). In the ileum, the mRNA expression of FXR and FGF19 was downregulated (*p* < 0.05). Regarding BA transport, the mRNA expression of organic anion transport peptides (OATPs) in the livers of SPs was significantly higher than that of NPs (*p* < 0.05), while the bile salt output pump (BSEP) did not change significantly (*p* > 0.05). The expression level of the organic anion transporter (OST)-β gene in the ileum decreased (*p* < 0.05), and OSTα showed no significant difference (*p* > 0.05). The Spearman correlation analysis suggested that the expression of the BA receptor TGR5 and FXR was highly correlated with CYP27A1 (R = 0.63, 0.86, Figure 2, the corresponding *p*-value as shown in Table S2). These results imply that the hepatic–intestinal circulation of BAs and the synthesis of BAs in piglets are enhanced after birth and may promote one another.

### 3.5. Correlation between BAs and Intestinal Barrier Development

**Figure 3:**
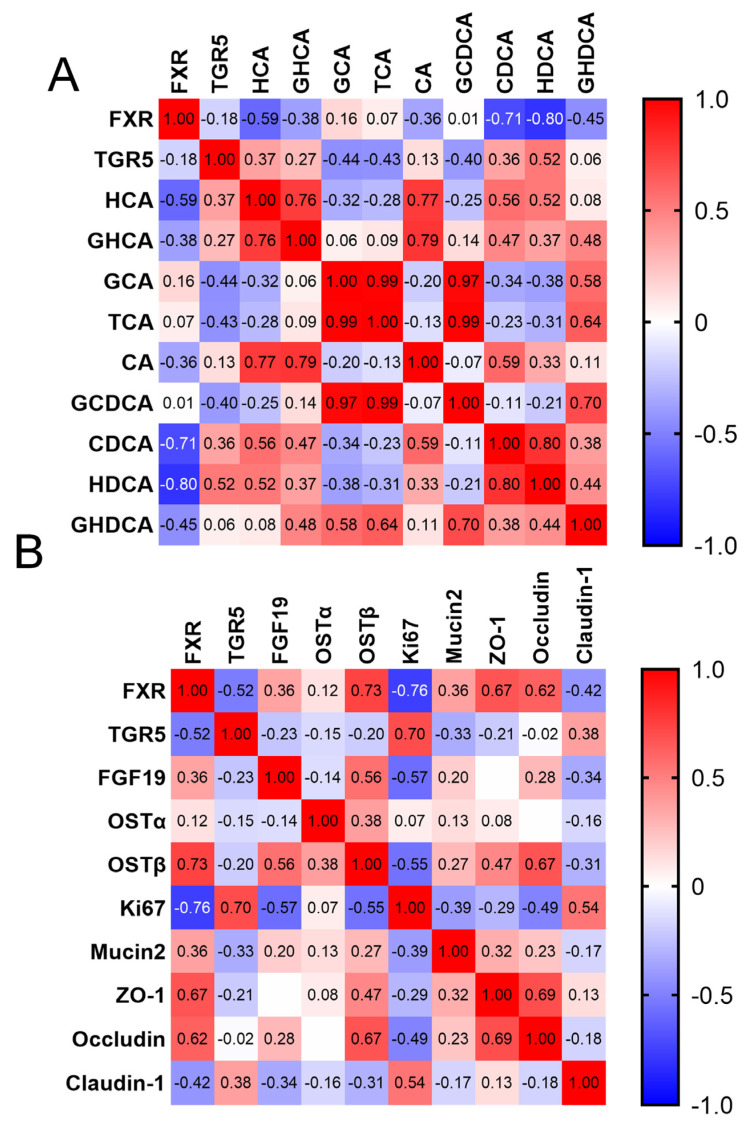
The correlation analysis between ileal mRNA expression and bile acids in newborn piglets (NPs) and suckling piglets (SPs): (**A**) correlation analysis between gene expression and bile acid content. (**B**) Correlation analysis between gene expression of bile acid receptors and transport gene expression of intestinal barrier function. Spearman’s method was used to analyze the correlation between variables (*n* = 12).

According to the mRNA expression, TGR5 in the ileum was significantly upregulated in suckling piglets, while FXR was downregulated significantly (*p* < 0.05, Table 6). Spearman correlation analyses were performed to explore the expression correlation between BA receptor gene expression and BA concentration (Figure 3A, the corresponding *p*-value as shown in Table S3). It was found that FXR showed a strong negative correlation with HCA, CDCA, and HDCA (R = −0.59, −0.71, and −0.80, respectively), while TGR5 only demonstrated a medium correlation with HDCA (R = 0.52). The results revealed that Ki-67 and claudin-1 were upregulated, while occludin was significantly downregulated (*p* < 0.05, Table 6). MUC2 and ZO-1 showed no significant changes (*p* > 0.05). Furthermore, gene expression related to the intestinal barrier was detected, and a correlation analysis was conducted (Figure 3B, the corresponding *p*-value as shown in Table S4). Ki-67 presented a medium positive correlation with TGR5 (R = 0.70, Figure 3B). ZO-1 and occludin displayed a strong correlation with FXR (R = 0.67, 0.62). Intriguingly, TGR5 and FXR exhibited a negative correlation (R = −0.52). These findings imply that the BA receptor TGR5 may modulate the proliferation of intestinal epithelial cells, while FXR regulates the formation of the intestinal barrier function. However, further exploration is required due to the complex composition of BAs in piglets.

## 4. Discussion

Due to changes in nutritional sources and environment before and after birth, the metabolic process of piglets experiences dramatic shifts before and during lactation [3,20]. Recent research reported that serum indices of globulin, albumin, cholesterol, HDL, and LDL in piglets tend to stabilize during 7–14 days of lactation and show no significant differences from those at weaning (i.e., the 21st day after birth) [3]. In line with previous studies, the present results suggest that the contents of albumin, globulin, glucose, total cholesterol, HDL, and LDL in piglets’ sera were significantly upregulated on day 14 compared with the newborn piglets, while the ALP contents were downregulated. Therefore, glycolipid metabolism and protein metabolism in piglets are highly activated within the first 14 days after birth. This stage is crucial for piglets’ development due to their growth performance being impaired after weaning.

BAs not only enhance fat absorption and regulate glucose and lipid metabolism but also act as essential signaling molecules in controlling intestinal epithelial homeostasis [21]. Nevertheless, BA metabolism and the principles of hepatic–intestinal circulation in piglets remain unclear. The present study found that the BA contents in suckling piglets’ blood increased by approximately 50%, which is consistent with the increase in lipid contents described here. Simultaneously, the present study observed an increased proportion of secondary BAs and free BAs in serum, which may suggest bacterial colonization and cross-talk in piglets’ intestines after birth, according to previous viewpoints [7]. Moreover, enhanced BA synthesis in piglets may primarily be modulated by the upregulation of CYP27A1, a key enzyme in the alternative BA synthesis pathway, rather than by CYP7A1 expression. This finding implies that porcine-specific BAs, such as HCA, may be primarily induced by the alternative synthesis pathway rather than the classical pathway, confirming the views of earlier researchers [22]. Previous studies have shown that BA synthesis can be significantly promoted by activating TGR5 in the liver [19]. It has also been demonstrated that downregulating FGF15/19 could enhance BA synthesis in mice and humans [23]. Similarly, in this study, TGR5 was significantly upregulated in the liver and ileum, while FGF19 was significantly downregulated in the ileum. This finding reveals that piglets may suppress ileal FGF19 expression and increase BA synthesis by activating TGR5 in the liver after birth. Concerning BA transportation, the liver membrane transport proteins OATP and BSEP primarily function as carriers for BA influx from the blood and BA efflux from the liver, respectively [21,24]. Furthermore, the increased OATP gene expression observed in piglets’ livers in this study suggests that the liver’s BA reabsorption capacity is enhanced during piglets’ growth.

Similarly, the morphology of the jejunum and ileum in piglets tends to mature during 7–14 days after birth, as reported in another study [3]. A common observation is that the villus height of the small intestine and the ratio of villus height to crypt depth decrease 7–14 days after birth. The ileum also exhibits a slight reduction in the number of goblet cells [4]. In this research, genes related to ileal cell proliferation and barrier function in piglets were examined to elucidate the regulatory effect of BAs on ileal epithelial cells. The results demonstrate that the gene expression of Ki-67 was upregulated, while occludin was significantly downregulated, and mucin2 and ZO-1 expression decreased to some extent, which partially aligns with the previous finding of a decrease in goblet cells and villus height [4]. This study’s findings suggest that the contents of secondary BAs, such as HDCA, LCA, and DCA, increased significantly after birth. The formation of secondary bile acids is regulated by the intestinal microbiota. For instance, key enzymes such as bile salt hydrolases, which can promote the conversion of conjugated bile acids to free bile acids, have been found in almost all bacterial phyla and archaeal systems [25]. In the intestines of growth-retarded piglets, the abundance of *Bacteroidia* was positively correlated with the contents of glycine-conjugated bile acids (such as GHCA), while the abundance of *Lactobacillus* was negatively correlated with GHCA and positively correlated with HCA levels [15]. Additionally, certain bacterial groups, such as *Clostridium scindens*, *Clostridium hylemonae*, *Clostridium perfringens*, and *Peptostreptococcus hiranonis*, have been found to regulate the epimerization of bile acids. For example, *Ruminococcus gnavus*, *Clostridium absonum*, *Stenotrophomonas maltophilia*, and *Collinsella aerofaciens* can regulate the conversion of CDCA to UDCA or LCA [25]. The structure of the intestinal microbiota in piglets is influenced by their age and diet composition. Studies have shown that the most significant changes in the intestinal microbiota occur between 0 days and 2 weeks after birth. After weaning, the source of nutrition for piglets shifts from maternal milk to solid feed, which also leads to further changes in the intestinal microbiota [26]. LCA and DCA have been reported to substantially inhibit the intestinal barrier function of piglets [12,27]. However, CDCA was found to alleviate the intestinal epithelial barrier injury induced by LPS [11]. Furthermore, the terminal ileum is considered to be the primary site of BA reabsorption, where approximately 95% of BAs are absorbed into the blood [28]. These pieces of evidence imply that morphological changes in the ileal epithelium may be closely related to BAs.

BA receptors are the primary pathway through which BAs regulate intestinal bioactivities. Previous research indicated that TCDCA suppressed FXR expression in FHs 74 Int cells (a human-derived intestinal cell line) and murine intestinal cells [23]. HDCA activates FXR and inhibits cell proliferation [13]. Conversely, research has demonstrated that CDCA can activate FXR and promote intestinal barrier damage repair [11]. Other research has shown that low doses of HDCA can activate TGR5 in STC-1 cells, while high doses of HDCA can activate FXR [29]. Moreover, there may be an interaction between TGR5 and FXR expression, with TGR5 activation potentially inhibiting FXR expression in the liver [19]. Whether this phenomenon exists in the intestine remains unclear. In this study, HDCA and CDCA were strongly negatively correlated with FXR expression, while they displayed moderate and weak correlations with TGR5 expression, respectively. These findings suggest that the downregulation of FXR expression relative to TGR5 might be the key reason for the upregulation of Ki-67 expression. However, the activation or inhibition between BAs and BA receptors may vary depending on the structure, concentration level, and tissues and organs of BAs, necessitating further investigation.

BA receptors are critical for the proliferation and barrier formation of intestinal cells. The present data reveal that TGR5 expression is significantly upregulated in the ileal epithelium of piglets, showing a positive correlation with Ki-67. Concurrently, FXR expression is significantly downregulated and negatively correlated with Ki-67. In other words, TGR5 may stimulate the proliferation of intestinal epithelial cells. Prior research has indicated that TGR5 expression is markedly downregulated in the tissues of mice and humans with IBD, and activating TGR5 can promote the regeneration of the intestinal epithelium and the formation of tight junctions [17,30,31]. FXR, an essential BA receptor, plays a pivotal role in regulating cell proliferation. Previous studies have shown that activating FXR can inhibit the proliferation of intestinal epithelial cells and intestinal stem cells. For instance, DCA and tauro-β-muricholic acid can induce the proliferation of intestinal stem cells by antagonizing FXR activity [32]. Conversely, FXR activation has been found to significantly suppress IPEC-J2 proliferation [13]. Similar phenomena have been observed in cancer cells from different tissues. Depending on the FXR expression levels, agonists or antagonists can be employed for cancer treatment [33]. This implies that FXR’s regulatory activity on cell proliferation may be associated with factors such as species and tissue. Therefore, in this study, the upregulation of TGR5 and downregulation of FXR might have been key factors contributing to the enhanced proliferation of intestinal cells in piglets after birth. However, the exact mechanism through which metabolic changes in BAs affect the development of the intestinal epithelium during piglet’s growth and development after birth remains to be elucidated.

In summary, BA synthesis in piglets is highly activated after birth. Nevertheless, the reabsorption of BAs at the end of the ileum may be the main reason for the decrease in goblet cell numbers and the expression of mucin and tight junction proteins within 14 days after birth. The renewal and development of the intestinal epithelium may be maintained by different BAs through a variety of pathways.

## 5. Conclusions

The results of this study indicate that the synthesis and metabolism of BAs (particularly HCA and its derivatives) and the liver’s reabsorption capacity are enhanced within 14 days after birth. BAs such as HCA, CDCA, and HDCA may contribute to the upregulation of TGR5 expression and the downregulation of FXR expression, potentially triggering intestinal cell proliferation and influencing the formation of barrier functions. The present findings offer novel insights and perspectives for uncovering the development and renewal of the intestinal epithelium in piglets post-birth, while laying the foundation for the nutritional regulation of young animals during this stage.

## Figures and Tables

**Table 1:** Ingredients and chemical composition of the diets (as-fed basis, 1%).

Item	Content (Gestation)	Content (Lactation)
Ingredients		
Corn	51.85	53.10
Soybean meal	10.80	23.50
Wheat bran	19.70	9.75
Corn protein powder	1.50	1.50
Extruded Soybeans	8.00	2.00
Soybean oil	3.00	5.00
CaHPO4·2H_2_O	2.00	2.00
Limestone	1.35	1.35
Salt	0.30	0.30
Sodium sulfate	0.30	0.30
Lysine sulfate (70%)	0.20	0.20
Premix ^1^	1.00	1.00
Total	100.00	100.00
Nutrient composition ^2^		
DE, MCal/kg	3.24	3.41
CP	15.95	18.72
EE	6.98	7.06
CF	3.42	3.59
Ca	1.07	1.09
Lys	0.93	1.21
Met + Cys	0.54	0.64
Thr	0.58	0.75
Trp	0.19	0.24

^1^ The premix provided the following per kg of complete diet: VA 6, 000 IU; VD3 2, 100 IU; VE, 45 IU; VK3, 2.0 mg; thiamine, 3.5 mg; riboflavin, 7.0 mg; VB6, 7.5 mg; VB12, 0.05 mg; choline chloride 1, 250 mg; nicotinic acid, 25.0 mg; pantothenic acid, 15.0 mg; biotin, 0.5 mg; folic acid, 1.0 mg; Mn, 20.0 mg; Zn, 80.0 mg; Fe, 80 mg; Cu, 10.0 mg; I, 0.15 mg; Se, 0.25 mg. ^2^ Nutrient levels were calculated values, and the nutritive values referred to the Feed Composition and Nutritive Values of the China Feed-Database Information Network Center (http://www.chinafeeddata.org.cn/, accessed on 12 July 2021).

**Table 2:** The body weight changes in newborn and suckling piglets.

Items ^1^	Groups ^2^ NPs (d1)	Groups SPs (d14)	SEM	*p*-Value
ABW (kg)	1.47	3.48	0.09	<0.001
Litter weight (kg)	17.82	30.43	1.51	<0.001
ADG (kg)	-	0.14	0.02	-
Backfat thickness of sows (mm)	19.67	17.92	0.88	0.378

^1^ ABW: average body weight of piglets, ADG: average daily weight gain of piglets. ^2^ NPs: newborn piglets, SPs: 14-day-old suckling piglets; *n* = 12 replicates per group.

**Table 3:** The serum parameters in piglets.

Items ^1^	Groups ^2^ NPs (d1)	Groups SPs (d14)	SEM	*p*-Value
TP (g/L)	15.86	31.16	2.39	<0.001
ALB (g/L)	6.27	23.33	2.18	<0.001
ALT (U/L)	7.96	16.70	1.42	0.001
AST (U/L)	22.21	39.08	4.28	0.046
ALP (U/L)	1024.18	598.75	198.50	0.024
BUN (mmol/L)	2.84	2.23	0.22	0.176
GLU (mmol/L)	1.93	5.19	0.53	0.002

^1^ TP: total protein, ALB: albumin, ALT: alanine aminotransferase, AST: aspartate aminotransferase, ALP: alkaline phosphatase, BUN: blood urea nitrogen, GLU: serum glucose. ^2^ NPs: newborn piglets, SPs: 14-day-old suckling piglets; *n* = 12 replicates per group.

**Table 4:** Differences in lipid and total bile acid contents in the serum and liver between NPs and SPs.

Items ^1^	Groups ^2^ NPs (d1)	Groups SPs (d14)	SEM	*p*-Value
Serum TG (mmol/L)	0.29	1.18	0.12	<0.001
Serum TC (mmol/L)	0.96	2.69	0.21	<0.001
Serum LDL-C (mmol/L)	0.38	1.37	0.14	<0.001
Serum HDL-C (mmol/L)	0.33	1.34	0.16	<0.001
Serum TBA (μmol/L)	14.15	33.88	4.44	0.026
Liver TG (mmol/g prot)	1.21	0.78	0.11	0.044
Liver TC (mmol/g prot)	0.41	0.39	0.02	0.487
Liver TBAs (μmol/g prot)	5.92	6.28	0.20	0.288

^1^ TG: triglyceride, TC: total cholesterol, LDL-C: low-density lipoprotein cholesterol, HDL-C: high-density lipoprotein cholesterol, TBAs: total bile acids. ^2^ NPs: newborn piglets, SPs: 14-day-old suckling piglets; *n* = 12 replicates per group.

**Table 5:** Serum bile acid contents of piglets (nmol/L).

Items ^1^	Groups ^2^ NPs (d1)	Groups SPs (d14)	SEM	*p*-Value
HCA	6.67	11,137.29	1939.51	0.002
GHCA	1030.56	3512.35	609.82	0.041
CA	18.73	145.73	30.78	0.004
TCA	39.26	6.70	15.02	0.818
GCA	130.12	20.52	40.21	0.093
CDCA	64.16	2252.73	405.32	0.002
TCDCA	524.24	428.04	168.80	0.937
GCDCA	852.88	410.58	512.82	0.818
HDCA	63.36	3146.25	543.81	0.002
THDCA	142.14	874.12	182.89	0.009
GHDCA	488.31	769.68	144.40	0.180
DCA	2.24	57.03	15.48	0.002
LCA	10.97	56.72	11.85	0.041
7-ketoLCA	12.92	47.82	10.98	0.041
3-DHCA	-	46.02	8.35	0.002
12-DHCA	-	63.65	26.42	0.015
7-KHCA	-	9.30	2.11	0.065
isoLCA	-	8.152	2.37	0.065
αMCA	-	27.20	5.40	0.002
UDCA	-	11.34	3.04	0.015
PBA	2666.60	17,941.12	2705.34	0.004
SBA	719.93	5090.08	780.78	0.004
FBA	179.03	17,009.20	1379.50	0.002
CBA	3207.50	6021.96	1368.83	0.240

^1^ PBA: primary bile acid, SBA: secondary bile acid, FBA: free bile acid, CBA: conjugated bile acid. PBAs include HCA, GHCA, CA, TCA, GCA, CDCA, TCDCA, GCDCA, and αMCA. SBAs include HDCA, THDCA, GHDCA, DCA, LCA, 7-ketoLCA, 3-DHCA, 12-DHCA, 7-KHCA, isoLCA, and UDCA. FBAs include HCA, CA, CDCA, HDCA, DCA, LCA, 7-ketoLCA, 3-DHCA, 12-DHCA, 7-KHCA, isoLCA, αMCA, and UDCA. CBAs include GHCA, TCA, GCA, TCDCA, GCDCA, THDCA, and GHDCA. ^2^ NPs: newborn piglets, SPs: 14-day-old suckling piglets. “-”: Not detected; data are shown as mean ± SEM; *n* = 6 replicates per group.

**Table 6:** The mRNA expression levels related to bile acid synthesis, hepatic–intestinal circulation, and barrier function in the ileum and liver.

Biological Function	Items ^1^	Groups ^2^ NPs (d1)	Groups SPs (d14)	SEM	*p*-Value
Bile acid synthesis	Liver-CYP7A1	1.00	1.27	0.29	0.657
	Liver-CYP8B1	1.00	0.60	0.12	0.101
	Liver-CYP27A1	1.00	1.98	0.27	0.003
	Liver-BSEP	1.00	1.26	0.19	0.297
	Liver-OATP	1.00	6.58	0.69	0.000
	Ileum-OSTα	1.00	1.14	0.14	0.623
	Ileum-OSTβ	1.00	0.45	0.11	0.008
Bile acid hepatic–intestinal circulation and bile acid receptor	Liver-FXR	1.00	1.32	0.08	0.051
	Liver-TGR5	1.00	1.49	0.12	0.044
	Liver-SHP	1.00	0.78	0.08	0.355
	Liver-FGFR4	1.00	1.98	0.17	0.003
	Liver-FGF19	1.00	0.66	0.12	0.169
	Ileum-FXR	1.00	0.35	0.08	<0.001
	Ileum-TGR5	1.00	2.24	0.23	0.005
	Ileum-FGF19	1.00	0.17	0.15	0.004
	Ileum-Ki-67	1.00	5.39	0.53	<0.001
	Ileum-MUC2	1.00	0.93	0.16	0.11
	Ileum-ZO-1	1.00	0.71	0.07	0.16
Intestinal barrier function	Ileum-Occludin	1.00	0.42	0.10	0.005
	Ileum-Claudin1	1.00	1.51	0.12	0.04

^1^ CYP7A1: cytochrome P450 family 7 subfamily A member 1, CYP8B1: cytochrome P450 family 8 subfamily B member 1, CYP27A1: cytochrome P450 family 27 subfamily A member 1, BSEP: bile salt output pump, OATP: organic anion transport peptide, OSTα/β: organic anion transporter, FXR: farnesoid X receptor, TGR5: Takeda G-coupled protein receptor 5, SHP: small heterodimer partner, FGFR4: fibroblast growth factor receptor 4, FGF19: fibroblast growth factor 19. ^2^ NPs: newborn piglets, SPs: 14-day-old suckling piglets; *n* = 12 replicates per group.

## Data Availability

The data supporting the findings of the present study are available from the corresponding author upon reasonable request.

## Supplementary Materials

The following supporting information can be downloaded at https://www.mdpi.com/article/10.3390/ani14020287/s1: Tables S1–S4. List of primers used for qRT-PCR analysis and *p*-values indicated for correlations (corresponding Figure 2 and Figure 3A,B).
