# Supplementary material for: Bile Acid Metabolic Profiles and Their Correlation with Intestinal Epithelial Cell Proliferation and Barrier Integrity in Suckling Piglets
Source: Animals (Basel). 2024 Jan 17;14(2):287. doi: 10.3390/ani14020287 (PMC10812516; doi:10.3390/ani14020287)
Supplement: Supplementary file 1 [file animals-14-00287-s001.zip › animals-2770375-supplementary.pdf]

Table S1 List of primers used for qRT-PCR analysis

Genes	Primers	Sequences (5′–3′)	Product length, bp
β-actin	Forward	CGTTGGCTGGTTGAGAATC	132
	Reverse	CGGCAAGACAGAAATGACAA	
CYP7A1	Forward	TTCCTTCTGCTACCGAGTGATG	102
	Reverse	AGGTTGTTTAGGATGAGTGCTTTC	
CYP8B1	Forward	GCAGGCAAGAAGATCCACCACTAC	112
	Reverse	TGACCATGAGCAGCACAAAGAGC	
CYP27A1	Forward	AGTTGGTGGCTTCCTCTTTCC	141
	Reverse	TTGAGAGCATCCGTCTGGTTC	
FXR	Forward	AGGACCGAGAGGCAGTAGAG	148
	Reverse	GCGTGGTGATGGTTGAATGTC	
TGR5	Forward	TGCTGTCCCTCATCTCATTGG	80
	Reverse	TGTGTAGCGATGATCACCCAG	
SHP	Forward	CGCTTGGCTCGTGTTCTCCTC	95
	Reverse	ACATCTCCAATGACAGGGCGAAAG	
FGFR4	Forward	GCCTGCTGGGAACACTGTCAAG	120
	Reverse	AGCCTAATGCCTCCAATGCGATTC	
FGF19	Forward	AAGATGCAAGGGCAGACTCA	101
	Reverse	AGATGGTGTTTCTTGGACCAGT	
BSEP	Forward	GCCTGGTCATAGTGTGCTTCTTCC	119
	Reverse	AGTAATCTGTGCGGCGACCTCTAG	
OATP	Forward	GAAATGTGGTGAGTCAGGGGCATG	87
	Reverse	CCTCTTAGTGCTGCTGGCAATCC	
OSTα	Forward	GGTGAGCAGAACATAGGAGCCAAG	122
	Reverse	AAGGGAGGCGAACAAGCAATCTG	
OSTβ	Forward	GTGGCTGTGGTGGTCGTGATAAG	110
	Reverse	AGGTAGAGGGCTTCTGGATGCTG	

ZO-1	Forward	GTATCCACTCTGCTAATGCTTCTG	138
	Reverse	AAGGCTCTGACCGCTGAT	
Occludin	Forward	GTGGGACAAGGAACGTATTTATG	115
	Reverse	TCTCTCCGCATAGTCCGAAA	
Claudin-1	Forward	CCCGTGCCTTGATGGTAAT	142
	Reverse	AGAAAGATCACTCCCCCAATG	
Ki-67	Forward	AGAACAAAGCCGTCAAGCG	105
	Reverse	TGCTGAACTGGTTCTGAAGGAT	
MUC2	Forward	GGTCATGCTGGAGCTGGACAGT	181
	Reverse	TGCCTCCTCGGGGTCGTCAC	

Table S2. The significance of the correlation between liver gene expression ***(p-values)****.*

	FXR	TGR5	SHP	FGFR4	FGF19	CYP7A1	CYP8B1	CYP27A1	BSEP	OATP
FXR		0.001	0.484	0.000	0.251	0.040	0.336	0.000	0.000	0.001
TGR5	0.001		0.278	0.001	0.023	0.046	0.034	0.001	0.000	0.009
SHP	0.484	0.278		0.943	0.723	0.957	0.797	0.943	0.379	0.302
FGFR4	0.000	0.001	0.943		0.304	0.120	0.553	0.000	0.000	0.000
FGF19	0.251	0.023	0.723	0.304		0.359	0.000	0.304	0.090	0.636
CYP7A1	0.040	0.046	0.957	0.120	0.359		0.264	0.120	0.076	0.037
CYP8B1	0.336	0.034	0.797	0.553	0.000	0.264		0.553	0.164	0.457
CYP27A1	0.000	0.001	0.943	0.000	0.304	0.120	0.553		0.000	0.000
BSEP	0.000	0.000	0.379	0.000	0.090	0.076	0.164	0.000		0.008
OATP	0.001	0.009	0.302	0.000	0.636	0.037	0.457	0.000	0.008	

Table S3. The significance of the correlation between bile acid receptor and bile acid ***(p-values)***.

	FXR	TGR5	HCA	GHCA	GCA	TCA	CA	GCDCA	CDCA	HDCA	GHDCA
FXR		0.578	0.042	0.223	0.630	0.835	0.256	0.985	0.009	0.002	0.147
TGR5	0.578		0.240	0.398	0.153	0.161	0.681	0.193	0.250	0.080	0.842
HCA	0.042	0.240		0.004	0.303	0.384	0.003	0.435	0.057	0.081	0.797
GHCA	0.223	0.398	0.004		0.858	0.770	0.002	0.674	0.125	0.240	0.114
GCA	0.630	0.153	0.303	0.858		0.000	0.527	0.000	0.276	0.223	0.047
TCA	0.835	0.161	0.384	0.770	0.000		0.687	0.000	0.478	0.332	0.026
CA	0.256	0.681	0.003	0.002	0.527	0.687		0.824	0.044	0.294	0.725
GCDCA	0.985	0.193	0.435	0.674	0.000	0.000	0.824		0.736	0.506	0.011
CDCA	0.009	0.250	0.057	0.125	0.276	0.478	0.044	0.736		0.002	0.220
HDCA	0.002	0.080	0.081	0.240	0.223	0.332	0.294	0.506	0.002		0.155
GHDCA	0.147	0.842	0.797	0.114	0.047	0.026	0.725	0.011	0.220	0.155	

Table S4. The significance of the correlation between Intestinal gene expression ***(p-values).***

	FXR	TGR5	FGF19	OSTα	OSTβ	Ki67	Mucin2	ZO-1	Occludin	Claudin-1
FXR		0.010	0.086	0.588	0.000	0.000	0.079	0.000	0.001	0.042
TGR5	0.010		0.269	0.495	0.339	0.000	0.118	0.324	0.933	0.070
FGF19	0.086	0.269		0.527	0.005	0.003	0.361	0.994	0.192	0.101
OSTα	0.588	0.495	0.527		0.067	0.755	0.537	0.721	0.982	0.448
OSTβ	0.000	0.339	0.005	0.067		0.005	0.195	0.021	0.000	0.143
Ki67	0.000	0.000	0.003	0.755	0.005		0.058	0.173	0.016	0.006
Mucin2	0.079	0.118	0.361	0.537	0.195	0.058		0.129	0.288	0.430
ZO-1	0.000	0.324	0.994	0.721	0.021	0.173	0.129		0.000	0.538
Occludin	0.001	0.933	0.192	0.982	0.000	0.016	0.288	0.000		0.410
Claudin-1	0.042	0.070	0.101	0.448	0.143	0.006	0.430	0.538	0.410	
